# ﻿Polynema (*Polynema*) Haliday (Hymenoptera, Mymaridae) of Xinjiang: descriptions of three new species, with eleven new records from China

**DOI:** 10.3897/zookeys.1251.161229

**Published:** 2025-09-04

**Authors:** Zhulidezi Aishan, Jin-Ling Wang, Sharengaowa, Serguei V. Triapitsyn

**Affiliations:** 1 College of Life Science and Technology, Xinjiang University, Urumqi, Xinjiang, 830017, China Xinjiang University Urumqi China; 2 Xinjiang Key Laboratory of Biological Resources and Genetic Engineering, Urumqi, Xinjiang, 830017, China Xinjiang Key Laboratory of Biological Resources and Genetic Engineering Urumqi China; 3 Entomology Research Museum, Department of Entomology, University of California, Riverside, California, 92521, USA University of California Riverside United States of America

**Keywords:** Central Palearctic region, Chalcidoidea, fairyfly, key, taxonomy

## Abstract

Species of the nominate subgenus of *Polynema* Haliday (Hymenoptera: Mymaridae) in Xinjiang Uyghur Autonomous Region of China are reviewed. Three new taxa are described, P. (Polynema) breviscapus Wang & Aishan, **sp. nov.**, P. (Polynema) gongliuense Sharengaowa & Aishan, **sp. nov.**, and P. (Polynema) haloxyloniae Sharengaowa & Aishan, **sp. nov.** Eight species are newly recorded from China: P. (Polynema) antoniae (Soyka, 1950); P. (Polynema) atrosimile Soyka, 1956; P. (Polynema) bakkendorfi Hincks, 1950; P. (Polynema) bischoffi (Soyka, 1950); P. (Polynema) capillatum Soyka, 1956; P. (Polynema) elongatum Soyka, 1956; P. (Polynema) fumipenne Walker, 1846; and P. (Polynema) pusillum Haliday, 1833. Three additional species are tentatively identified: P. (Polynema) ? elegantissimum Soyka, 1956; P. (Polynema) ? gracile (Nees, 1834); and P. (Polynema) ? neofuscipes (Soyka, 1946). A key to the species of P. (Polynema) from Xinjiang, China is provided.

## ﻿Introduction

*Polynema* Haliday, 1833 (Hymenoptera: Mymaridae) is a cosmopolitan genus comprising 223 described species (UCD 2025) in three subgenera: P. (Polynema) Haliday, 1833, P. (Dorypolynema) Hayat & Anis, 1999, and P. (Doriclytus) Foerster, 1847.

Hayat and Anis (1999) defined the nominate subgenus P. (Polynema), describing three new species and recording seven known species from India. Huber and Bouček (2001) designated *Polynema flavipes* Walker, 1846, as the type species of *Polynema*, confirming its placement in the nominate subgenus. Triapitsyn and Fidalgo (2006) provided taxonomic notes on P. (Polynema) and P. (Doriclytus), establishing key diagnostic characteristics and clarifying the proper taxonomic position of some species. Triapitsyn and Berezovskiy (2007) determined that *Polynema gracile* (Nees, 1834) belongs to P. (Polynema), supported by Graham’s (1973) redescription and their own examination of other specimens. Triapitsyn (2021) described eight new species of P. (Polynema) from Australia and New Zealand, and Anwar et al. (2024) described three new species in the nominate subgenus from Saudi Arabia.

Research on P. (Polynema) in China has been limited. Here we review the species of P. (Polynema) from Xinjiang Uyghur Autonomous Region of China and provide a key to their separation based on females.

## ﻿Materials and methods

Specimens were sourced from the Entomology Collection at the College of Life Science and Technology, Xinjiang University, Urumqi, Xinjiang, China (**ICXU**); these were accumulated over the years by collecting with Malaise traps and by sweep netting. Most mymarid specimens in the collection are slide mounted. A small portion of the specimens are preserved in ethanol at -20 °C for long term preservation.

Photographs of the slide-mounted specimens were captured using a Nikon SMZ25 (Nikon, SMZ25, Japan) system and the NIS-Elements software through focus stacking, and processed with Adobe Photoshop (Adobe, Photoshop CC). Measurements of slide-mounted specimens were taken using a Nikon compound microscope (Nikon, E200MV, Japan) with an ocular micrometer at 100×, 200×, or 400× magnification. Measurements are presented in micrometers (µm) or as ratios.

Morphological terms follow Triapitsyn (2018, 2021). Abbreviations used: **F** = funicle segments of the female antenna or flagellomeres of the male antenna; **mps** = multiporous plate sensillum or sensilla on the antennal flagellar segments (= longitudinal sensillum or sensilla) (Triapitsyn and Aquino 2010).

## ﻿Results

### ﻿ Polynema (Polynema)

Taxon classificationAnimaliaHymenopteraMymaridae

Haliday, 1833

44F0627A-60DE-5EE9-8DAB-9F64D6E5633D

Polynema Haliday, 1833a: 268; 1833b: 347. Type species: Polynema flavipes Walker, 1846; by subsequent designation by Huber and Bouček 2001: 281.
Polynema (Polynema) Haliday: Triapitsyn and Fidalgo 2006: 57 (key to subgenera, discussion).

#### Diagnosis.

**Figure 1.**
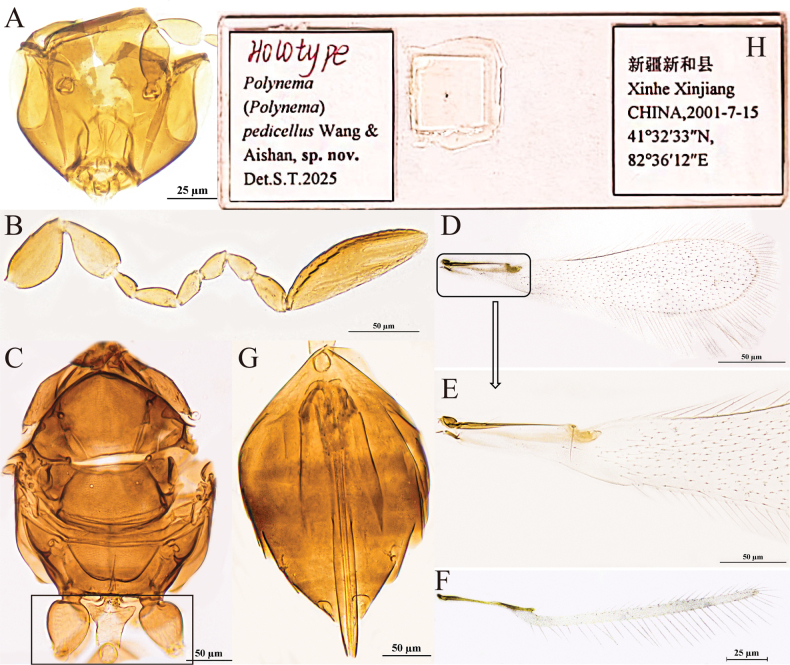
P. (Polynema) breviscapus sp. nov. ♀ (holotype). A. Head in frontal view; B. Antenna; C. Mesosoma; D. Forewing; E. Submarginal vein; F. Hind wing; G. Metasoma; H. Glass slide.

**Figure 2.**
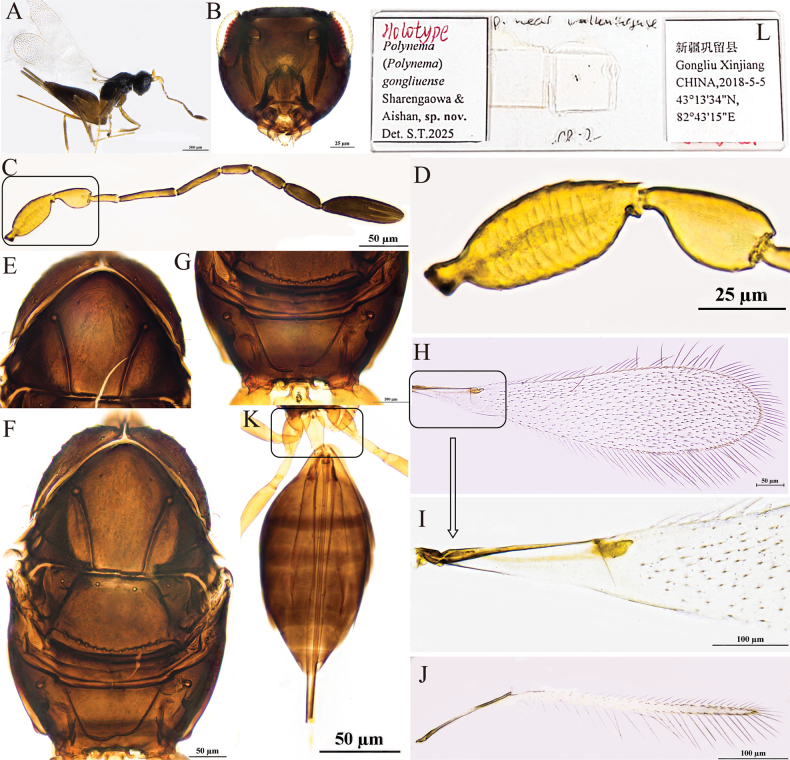
P. (Polynema) gongliuense sp. nov. ♀ (holotype). A. Body; B. Head in frontal view; C. Antenna; D. Scape and pedicel; E. Pronotum; F. Mesosoma; G. Propodeum; H. Forewing; I. Submarginal vein; J. Hind wing; K. Metasoma; L. Glass slide.

**Figure 3.**
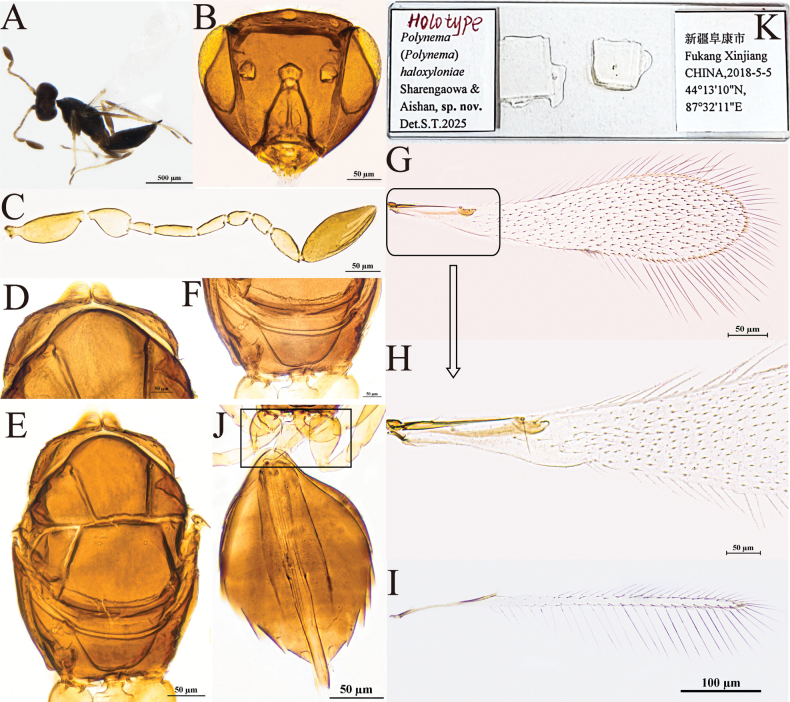
P. (Polynema) haloxyloniae sp. nov. ♀ (holotype). A. Body; B. Head in frontal view; C. Antenna; D. Pronotum; E. Mesosoma; F. Propodeum; G. Forewing; H. Submarginal vein; I. Hind wing; J. Metasoma; K. Glass slide.

Body color generally pale to dark brown, antenna (Figs 2A, 8A, 14A) with scape and pedicel generally paler than the body. Face (Figs 1A, 2B, 3B) without a pit next to each torulus. Most male genitalia with digitus bearing a strongly curved claw with one or two distinct denticles on its concave margin.

### ﻿Key to species of Polynema (Polynema) in Xinjiang, China (females)

**Table:** 

1	Scape inner surface with cross-ridges (Fig. 2D)	**2**
–	Scape inner surface without cross-ridges (Fig. 3C)	**4**
2	Clava with 7 mps; pronotum with 4 setae on each side along anterior margin; propodeum with a short median carina; forewing with hypochaeta not touching posterior margin of forewing	**3**
–	Clava with 6 mps; pronotum with 3 setae on each side along anterior margin; propodeum (Fig. 2G) without a median carina; forewing with hypochaeta touching posterior margin of forewing (Fig. 2I)	**P. (Polynema) gongliuense Sharengaowa & Aishan, sp. nov.**
3	Forewing with dark microtrichia on disc beginning well beyond apex of stigmal vein (Fig. 5D); petiole without vertical striations; ovipositor ≤ 1.4× length of metatibia	**P. (Polynema) atrosimile Soyka**
–	Forewing with dark microtrichia on disc beginning behind stigmal vein (Fig. 13D); petiole in dorsal view with inconspicuous transverse striations (Fig. 13E); ovipositor ≥ 1.6× length of metatibia	**P. (Polynema) ? neofuscipes (Soyka)**
4	Scape, excluding radicle, longer than pedicel; scutellum posteriorly with a transverse row of small foveae; propodeum without median carina or with a short median	**5**
–	Scape, excluding radicle, as long as pedicel (Fig. 1B); scutellum posteriorly without a transverse row of small foveae (Fig. 1C); propodeum posteriorly with inverted “Y” shaped median carina (Fig. 1C)	**P. (Polynema) breviscapus Wang & Aishan, sp. nov.**
5	Ovipositor slightly exserted beyond gastral apex; scape less than 4× as long as wide	**6**
–	Ovipositor not exserted beyond gastral apex (Fig. 12D); scape 5× as long as wide (Fig. 12A)	**P. (Polynema) ? gracile (Nees)**
6	Hypochaeta not extending to posterior margin of forewing	**7**
–	Hypochaeta extending to posterior margin of forewing (Fig. 7D)	**P. (Polynema) bischoffi (Soyka)**
7	F1 and F4 subequal in length and both the shortest funiculars; F2 the longest funicular; pronotum along anterior margin with 4 setae on each side	**8**
–	F1 the shortest funicular (Fig. 3C); F2 and F6 subequal in length and both the longest funiculars; pronotum along anterior margin with 6 setae on each side	**P. (Polynema) haloxyloniae Sharengaowa & Aishan, sp. nov.**
8	Clava shorter than combined length of 3 preceding flagellomeres; mesosoma smooth	**9**
–	Clava as long as combined length of 3 preceding flagellomeres (Fig. 14B); mesoscutum with conspicuous reticulate sculpture (Fig. 14C)	**P. (Polynema) pusillum Haliday**
9	Scape ≥ 1.6× as long as pedicel; F2 the longest funicular; clava shorter than combined length of 3 preceding flagellomeres	**10**
–	Scape ≤ 1.5× as long as pedicel; F6 the longest funicular; clava longer than combined length of 3 preceding flagellomeres (Fig. 9B)	**P. (Polynema) ? elegantissimum Soyka**
10	Clava with 6 mps	**11**
–	Clava with 7 mps	**12**
11	Forewing with longest marginal seta ≤ 0.6× maximum wing width (Fig. 4D); scape ≤ 3.0× as long as wide (including short radicle) (Fig. 4B); F5 and F6 subequal in length	**P. (Polynema) antoniae (Soyka)**
–	Forewing with longest marginal seta ≥ 1.1× maximum wing width (Fig. 8D); scape ≥ 3.7× as long as wide (including short radicle) (Fig. 8B); F5 ≤ 0.8× as long as F6	**P. (Polynema) capillatum Soyka**
12	Ovipositor ≤ 1.5× length of metatibia; forewing with dark setae on disk beginning well beyond apex of stigmal vein	**13**
–	Ovipositor ≥ 1.6× length of metatibia (Fig. 6E); forewing with dark setae on disc beginning behind stigmal vein (Fig. 6D)	**P. (Polynema) bakkendorfi Hincks**
13	Pronotum along anterior margin with 8 setae on each side (Fig. 10C); forewing ≥ 4.6× as long as wide(Fig. 10D); metacoxa measured in lateral view almost as long as petiole (Fig. 10E)	**P. (Polynema) elongatum Soyka**
–	Pronotum along anterior margin with 4 setae on each (Fig. 11C); forewing ≤ 4.1× as long as wide(Fig. 11D); metacoxa measured in lateral view ≤ 0.9× as long as petiole (Fig. 11E)	**P. (Polynema) fumipenne Walker**

### ﻿Descriptions of new species (in alphabetical order)

### ﻿ Polynema (Polynema) breviscapus

Taxon classificationAnimaliaHymenopteraMymaridae

Wang & Aishan sp. nov.

6A1E1B83-FF4A-5314-B8AA-C60E9E64F0E1

https://zoobank.org/F83AE017-1B1B-4E44-93B4-14B7B1917EFC

Fig. 1

#### Type material.

***Holotype***: • ♀ (ICXU) on slide (Fig. 1H): China, Xinjiang, Xinhe, 41°32'33"N, 82°36'12"E, 15.VII.2001, Wei Wu, sweeping. ***Paratypes*** (all in ICXU): China, Xinjiang: • 1 ♀, Jinghe, 44°36'03"N, 82°53'22"E, 15.VII.2001, Hongying Hu et al., sweeping; • 4 ♀♀, Xinhe, 41°32'38"N, 82°36'16"E, 1.VI.2001, Wei Wu, sweeping.

#### Diagnosis.

Antenna (Fig. 1B) with scape smooth, almost as long as pedicel; F2 as long as F6; clava longer than combined length of three preceding flagellomeres and longer than combined length of F1–F3, with eight mps. Mesosoma smooth; pronotum (Fig. 1C) with four setae on each side along anterior margin; scutellum (Fig. 1C) with a frenal row of foveae, they are distinctly wider and apparently shallower; propodeum (Fig. 1C) with a complete median carina shaped posteriorly as an inverted narrow Y-shape. Ovipositor (Fig. 1G) slightly exserted beyond gastral apex.

#### Description.

**Female** (holotype and paratypes). Body length 426–438 μm (*n* = 6). Head (Fig. 1A) in frontal view 0.6× (0.6–0.8×) as high as wide. Antenna (Fig. 1B) with scape smooth, 1.8× (1.8–1.9×) as long as wide (including short radicle), almost as long as pedicel; pedicel 2.1× (1.5–2.1×) as long as wide, 2.3× (1.9–2.4×) as long as F1; F1 the shortest funicular; F2 as long as F6 and both the longest funiculars; F4 and F5 subequal in length; F6 with one mps; clava 3.3× (2.9–3.7×) as long as wide, longer than combined length of three preceding flagellomeres and longer than combined length of F1–F3, with eight mps.

Mesosoma smooth, 1.2× (1.1–1.3×) as long as wide; pronotum (Fig. 1C) divided mediolongitudinally, with four setae on each side along the anterior margin; mesoscutum (Fig. 1C) 0.5× (0.5–0.6×) as long as wide, longer than scutellum; scutellum (Fig. 1C) 0.6× (0.5–0.6×) as long as wide, with a frenal row of foveae, they are distinctly wider and apparently shallower; propodeum (Fig. 1C) with a complete median carina shaped posteriorly as an inverted narrow Y-shape. Forewing (Fig. 1D) 4.1× (4.1–4.4×) as long as wide; disc hyaline, densely setose beyond venation, with discal setae originating behind stigmal vein (Fig. 1E), many of them 5.3–7.2 μm long; longest marginal seta 1.0× (0.6–1.0×) greatest width of forewing; Hind wing (Fig. 1F) 28.9× (27.2–31.4×) as long as wide; disc hyaline, with two rows of setae; longest marginal seta 5.8× (5.6–6.4×) greatest width of hind wing.

Metacoxa (Fig. 1C) smooth, in lateral view a little longer than petiole. Petiole (Fig. 1C) 1.3× (1.3–1.5×) as long as wide, in dorsal view with inconspicuous vertical striations and expanded basally. Ovipositor (Fig. 1G) 1.0× (1.0–1.1×) as long as gaster, slightly exserted by ~0.1× its own total length beyond gastral apex; ovipositor 1.7× (1.5–1.8×) length of mesotibia and 1.5× (1.3–1.5×) length of metatibia.

Measurements of the holotype (μm). Head height: width: 60: 101; mesosoma (in dorsal view) length: width: 115: 95; mesoscutum length: width: 41: 77; scutellum length: width: 30: 53; median carina length: 29; petiole length: width: 28: 22; gaster length: width: 130: 90; ovipositor length: 136; exserted part of ovipositor: 13; antennal segments length: width: scape: 26: 15; pedicel: 25: 12; F1: 11: 5; F2: 18: 6; F3: 11: 6; F4: 13: 7; F5: 12: 7; F6: 18: 8; clava: 56: 17; forewing length: width: 274: 66; longest marginal seta length: 65; discal setae length: 5–7; hind wing length: width: 231: 8; longest marginal seta length: 46; mesotibia length: 79; metatibia length: 89.

**Male.** Unknown.

#### Hosts.

Unknown.

#### Etymology.

The new species is a noun in apposition meaning “a short scape” in Latin.

#### Distribution.

China (Xinjiang).

#### Comments.

In this new species, the scape is approximately as long as the pedicel, thus distinguishing it from most species within the subgenus in which the scape is usually longer than pedicel. The unique combination of the posterior margin of the scutellum with a frenal row of foveae that are distinctly wider and apparently shallower and the median carina being complete, forming an inverted Y-shape, separates it from all the other described species in the subgenus.

### ﻿ Polynema (Polynema) gongliuense

Taxon classificationAnimaliaHymenopteraMymaridae

Sharengaowa & Aishan sp. nov.

A6AD015D-C505-5332-9246-02ADA1EC8C7A

https://zoobank.org/4519E083-7390-4E48-84EE-0B5452E129D0

Fig. 2

#### Type material.

***Holotype***: • ♀ (ICXU) on slide (Fig. 2L): China, Xinjiang, Gongliu, 43°13'34"N, 82°43'15"E, 5.V.2018, Qin Li et al., sweeping. ***Paratypes*** (all in ICXU): China, Xinjiang: •1 ♀, Gongliu, 43°13'34"N, 82°43'15"E, 5.V.2018, Zhulidezi Aishan et al., sweeping; • 1 ♀, Yuli, 41°25'12"N, 86°14'24"E, 26.VII.2021, Zhulidezi Aishan et al., sweeping.

#### Diagnosis.

Antenna (Fig. 2C) scape with cross-ridges (Fig. 2C, D); F4 and F5 subequal in length and shorter than F6; F6 with one mps; clava shorter than combined length of three preceding flagellomeres and shorter than combined length of F1–F3, with six mps. Mesoscutum with conspicuous reticulate sculpture; pronotum (Fig. 2E) with three setae on each side along the anterior margin; propodeum (Fig. 2G) without median carina; forewing with hypochaeta extending to posterior margin of forewing (Fig. 2H, I). Ovipositor (Fig. 2K) exserted beyond gastral apex by 0.3× (0.2–0.3×) its own total length.

#### Description.

**Female** (holotype and paratypes). Body length 1560–1700 μm (*n* = 3). Body (Fig. 2A) black; scape and pedicel yellowish-brown, clava dark brown, legs pale brown.

Head (Fig. 2B) in frontal view 0.8× (0.8–1.2×) as high as wide. Antenna (Fig. 2C) scape with cross-ridges, 2.7× (2.0–2.7×) as long as wide (including short radicle), scape longer than pedicel, 1.7×(1.5–1.7) as long as pedicel; pedicel smooth, 1.9× (1.5–1.9×) as long as wide, 1.3× (1.3–1.6×) as long as F1; F1 the shortest and F2 the longest funiculars; F4 and F5 subequal in length and shorter than F6; F6 with one mps; clava 3.6× (3.4–3.6×) as long as wide, shorter than combined length of three preceding flagellomeres and shorter than combined length of F1–F3, with six mps.

Mesosoma 1.5× (1.5–1.7×) as long as wide; mesoscutum with conspicuous reticulate sculpture; scutellum and propodeum smooth; pronotum (Fig. 2E) divided mediolongitudinally, with three setae on each side along the anterior margin; mesoscutum (Fig. 2F) 0.7× as long as wide, longer than scutellum; scutellum (Fig. 2F) 0.6× (0.6–0.8×) as long as wide; frenum short, separated from scutellum by a row of small foveae; propodeum (Fig. 2G) without median carina. Forewing (Fig. 2H) 4.1× (4.1–4.3×) as long as wide; disc hyaline, densely setose beyond venation, with discal setae originating behind stigmal vein (Fig. 2I), many of them 6.1–7.3 μm long; forewing marginal hairs are significantly shorter;longest marginal seta 0.6× greatest width of forewing; hypochaeta extending to posterior margin of forewing (Fig. 2H, I). Hind wing (Fig. 2J) 50.6× (48.8–50.6×) as long as wide; disc hyaline, with two rows of setae; longest marginal seta 6.5× (6.1–6.5×) greatest width of hind wing.

Metacoxa (Fig. 2K) smooth, in lateral view ~0.9× as long as petiole; petiole (Fig. 2K) ~2.0× (2.0–2.5×) as long as wide, in dorsal view without vertical striations and expanded basally. Ovipositor (Fig. 2K) 1.3× (1.2–1.4×) as long as gaster, exserted beyond gastral apex by 0.3× (0.2–0.3×) its own total length; ovipositor 2.8× (2.5–2.8×) length of mesotibia and 2.4× length of metatibia.

Measurements of the holotype (μm). Head height: width: 93: 114; mesosoma (in dorsal view) length: width: 216: 148; mesoscutum length: width: 87: 125; scutellum length: width: 53: 88; median carina length: 20; petiole length: width: 69: 35; gaster length: width: 369: 181; ovipositor length: 480; exserted part of ovipositor: 123; antennal segments length: width: scape: 64: 24; pedicel: 38: 20; F1: 29: 8; F2: 60: 8; F3: 51: 7; F4: 34: 9; F5: 36: 11; F6: 41: 14; clava: 86: 24; forewing length: width: 656: 161; longest marginal seta length: 101; discal setae length: 6–7; hind wing length: width: 506: 10; longest marginal seta length: 65; mesotibia length: 173; metatibia length: 203.

**Male.** Unknown.

#### Hosts.

Unknown.

#### Etymology.

This species is named after its type locality.

#### Distribution.

China (Xinjiang).

#### Comments.

This species is relatively large in size (body length 1560–1700 μm). Its type specimens were collected in Gongliu County in Xinjiang at an altitude of 1220 m and in Yuli County at 890 m. The species has cross-ridges on the scape, a feature that is relatively rare within the subgenus. The marginal hairs on the forewings are significantly shorter than those of other, similar species.

Polynema (Polynema) gongliuense is very similar to the European P. (Polynema) valkenburgense Soyka in the following: the scape has distinct cross-ridges; F6 with one mps; the longest marginal setae on the forewing are shorter than the maximum wing width; and the ovipositor is distinctly exserted. The difference between them is as follows: the forewing 4.1× as long as wide in the former but 3.5× in the latter species; the longest marginal seta 0.6× greatest width of forewing in the former but 0.2× in the latter species; F3 shorter than scape in the former but subequal in length in the latter species. Propodeum without median carina in the former but with a short median carina in the latter species (Soyka 1950).

### ﻿ Polynema (Polynema) haloxyloniae

Taxon classificationAnimaliaHymenopteraMymaridae

Sharengaowa & Aishan sp. nov.

92D2CC01-AD07-5F12-A545-B33121FE8AD7

https://zoobank.org/BF06D01D-921D-4109-9B4C-48D3702D76BA

Fig. 3

#### Type material.

***Holotype***: • ♀ (ICXU) on slide (Fig. 3K): China, Xinjiang, Fukang, 44°13'10"N, 87°32'11"E, 5.V.2018, Hongying Hu et al., sweeping in *Haloxylon* forest. ***Paratypes*** (all in ICXU): China, Xinjiang: • 1 ♀, Altay City, 48°03'15"N, 86°26'35"E, 3.VIII.2007, Hongying Hu et al., sweeping in *Haloxylon* forest; • 1 ♀, Altay Prefecture, Bürjin, 38°06'15"N, 87°06'50"E, 7.VIII.2007, Hongying Hu et al., sweeping in *Haloxylon* forest; • 1 ♀, Fukang: 44°13'22"N, 87°32'11"E, 5.V.2017, Hongying Hu et al., sweeping in *Haloxylon* forest; • 1 ♀, 44°13'16"N, 87°33'18"E, 4.VI.2017, Hongying Hu et al., sweeping in *Haloxylon* forest.

#### Diagnosis.

Body dark brown (Fig. 3A); antenna (Fig. 3C) with scape smooth; F1 and F4 subequal in length and the shortest funiculars; clava shorter than combined length of F1–F3 but longer than combined length of 3 preceding flagellomeres, with seven mps. Mesosoma smooth; pronotum (Fig. 3D) with six setae on each side along the anterior margin; propodeum (Fig. 3F) with a short median carina. Forewing (Fig. 2G) 4.4× (4.0–4.4×) as long as wide. Ovipositor (Fig. 3J) slightly exserted beyond gastral apex.

#### Description.

**Female** (holotype and paratypes). Body length 860–930 μm (*n* = 5). Body dark brown (Fig. 3A), antenna brown except pedicel yellowish-brown, petiole pale brown, legs yellowish-brown.

Head (Fig. 3B) in frontal view 0.6× (0.6–0.8×) as high as wide. Antenna (Fig. 3C) with scape smooth, 2.6× (2.1–2.7×) as long as wide (including a short radicle); pedicel 1.6× (1.5–1.6×) as long as wide, with the same length as F2; F1 and F4 as long as and the shortest funiculars; F6 with one mps; clava 2.9× (2.6–2.9×) as long as wide, shorter than combined length of F1–F3 but longer than combined length of three preceding flagellomeres, with seven mps.

Mesosoma smooth, 1.4× (1.4–1.6×) as long as wide; pronotum (Fig. 3D) divided mediolongitudinally, with six setae on each side along the anterior margin; mesoscutum (Fig. 3E) wider than long; scutellum (Fig. 3E) 0.7× (0.7–0.8×) as long as wide, shorter than mesoscutum; frenum short, separated from scutellum by a row of small, inconspicuous foveae; propodeum (Fig. 3F) with a short median carina. Forewing (Fig. 3G) 4.4× (4.0–4.4×) as long as wide; disc hyaline, densely setose beyond venation, with discal setae originating behind apex of submarginal vein (Fig. 3H), many of them 9–17 μm long; longest marginal seta 0.9× (0.8–0.9×) greatest width of forewing; hypochchaeta not reaching posterior margin of forewing (Fig. 3H). Hind wing (Fig. 3I) 39× (39–42×) as long as wide; disc hyaline, with two rows of setae; longest marginal seta 6.4× (6.4–7.1×) greatest width of hind wing.

Metacoxa (Fig. 3J) smooth, in lateral view a little shorter than petiole. Petiole (Fig. 3J) ~1.8× (1.8–2.1×) as long as wide, in dorsal view with inconspicuous transverse striations, and expanded basally. Ovipositor (Fig. 3J) 1.0× (1.0–1.1×) as long as gaster, slightly exserted beyond gastral apex, 1.6× (1.6–2.0×) length of mesotibia and 1.7× (1.3–1.8×) length of metatibia.

Measurements of the holotype (μm). Head height: width: 89: 138; mesosoma (in dorsal view) length: width: 177: 131; mesoscutum length: width: 69: 113; scutellum length: width: 55: 77; median carina length: 6.5; petiole length: width: 48: 27; gaster length: width: 224: 142; ovipositor length: 232; exserted part of ovipositor: 30; antennal segments length: width: scape: 49: 19; pedicel: 32: 20; F1: 19: 7; F2: 32: 7; F3: 25: 7; F4: 19: 9; F5: 17: 11; F6: 27: 14; clava: 68: 23; forewing length: width: 478: 107; longest marginal seta length: 94; discal setae length: 4–7; hind wing length: width: 433: 11; longest marginal seta length: 70; mesotibia length: 141; metatibia length: 132.

**Male.** Unknown.

#### Hosts.

Unknown.

#### Etymology.

Named after the host plant genus, *Haloxylon* (Amaranthaceae).

#### Distribution.

China (Xinjiang).

#### Comments.

This species has thus far been recorded exclusively in the low-altitude desert regions of Xinjiang (376–525 m), with its host plants identified as *Haloxylon* spp. *Polynema haloxyloniae* has not been observed on any other vegetation types and is presumed to specifically parasitize eggs of hemipteran insects within this particular *Haloxylon* forest habitat.

##### ﻿Other newly recorded species of Polynema (Polynema) from China (in alphabetical order)

### ﻿ Polynema (Polynema) antoniae

Taxon classificationAnimaliaHymenopteraMymaridae

(Soyka, 1950)

FC99B948-2CDF-5A85-AF5E-67DED6A7C861

Fig. 4

Novickyella antoniae Soyka, 1950: 6.
Polynema antoniae (Soyka): Trjapitzin 1978: 536.

#### Material examined.

China, Xinjiang: • 1 ♀, Tekes, 43°09'19"N, 81°47'23"E, 9.VII.2021, Qin Li et al., sweeping; • 2♀♀, Yining, 43°42'03"N, 81°37'50"E, 8.VII.2021, Qin Li et al., sweeping. (All in ICXU).

#### Diagnosis.

**Figure 4.**
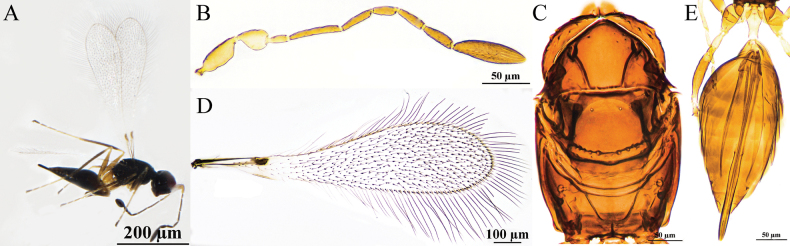
Polyneyma (Polynema) antoniae ♀ (Yining, Xinjiang, China). A. Body; B. Antenna; C. Mesosoma; D. Forewing; E. Metasoma.

**Female.** Body length 880–980 μm (*n* = 3). Body (Fig. 4A) dark brown, scape and pedicel yellowish-brown, clava dark brown, petiole yellowish-brown, legs pale brown. Antenna (Fig. 4B) with scape smooth, clava with six mps. Mesoscutum (Fig. 4C) smooth. Propodeum (Fig. 4C) with a short median carina. Forewing (Fig. 4D) with apical part distinctly broadened, dark discal cilia extending proximally to distinctly beyond stigma. Metacoxa (Fig. 4E) and petiole in lateral view subequal in length. Ovipositor (Fig. 4E) slightly exserted from the apex of gaster.

**Male.** Unknown.

#### Hosts.

Unknown.

#### Distribution.

China (Xinjiang); Austria.

#### Comments.

This species was collected in Xinjiang in both a lowland area of the Ili Valley and in the mid-elevation mountainous habitat in Tekes County. The morphology of this species is highly consistent with the type specimen described by Soyka in 1950, thus supporting their conspecificity. Shared characteristics include a predominantly dark brown body color; a similar forewing shape with the middle distinctly broadened and a length ~4.3–4.5× the width; the longest marginal setae reaching ~2/3 of the maximum wing width; the scape ~3× as long as wide and 1.6× as long as the pedicel; F2 being the longest flagellar segment and F1 the shortest. The ovipositor is clearly exserted. Differences lie in the fact that the original description did not specify the number of sensilla and lacked details on the mesosomal characteristics, whereas the modern description adds several structural features not previously recorded, such as a complete median longitudinal groove on the mesoscutum and a short median carina on the propodeum.

### ﻿ Polynema (Polynema) atrosimile

Taxon classificationAnimaliaHymenopteraMymaridae

Soyka, 1956

8BD1FB1B-65AE-53C9-9978-E2154AF22758

Fig. 5

Polynema atrosimilis Soyka, 1956: 41. Bouček 1977: 122.

#### Material examined.

China, Xinjiang: • 2♀♀, Gongliu: 43°13'33"N, 82°43'15"E, 10.VII.2021, Qin Li et al., sweeping; • 1 ♀, 43°16'19"N, 82°33'44"E, 10.VII.2021. Qin Li et al., sweeping; • 1 ♀, Xinyuan, 43°24'12"N, 82°33'45"E, 5.VII.2021. Qin Li et al., sweeping. (All in ICXU).

#### Diagnosis.

**Figure 5.**
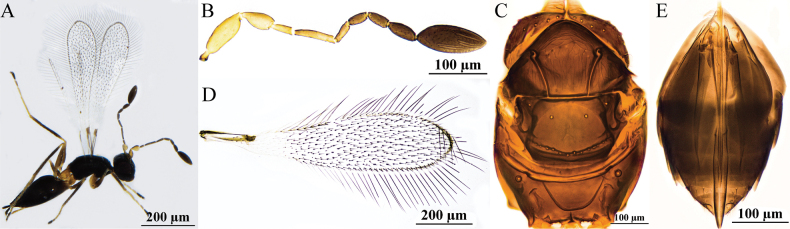
P. (Polynema) atrosimile ♀ (Gongliu, Xinjiang, China). A. Body; B. Antenna; C. Mesosoma; D. Forewing; E. Metasoma.

**Female.** Body length 790–800 μm (*n* = 4). Body (Fig. 5A) dark brown, scape and pedicel yellowish-brown, clava brown; petiole yellowish-brown, legs pale brown. Antenna scape (Fig. 5B) with indistinct cross-ridges; clava with seven mps. Mesoscutum (Fig. 5C) with conspicuous reticulate sculpture. Propodeum (Fig. 5C) with a short median carina. Forewing with longest marginal seta as long as greatest width of wing; dark microtrichia on disc beginning well beyond apex of stigma (Fig. 5D); Ovipositor (Fig. 5E) slightly exserted beyond apex of gaster.

**Male.** Unknown.

#### Hosts.

Unknown.

#### Distribution.

China (Xinjiang); Serbia.

#### Comments.

Polynema (Polynema) atrosimile is distributed in both mid-elevation Gongliu (1136 m) and lower-altitude Xinyuan (515 m). This species shares key morphological characteristics with the type species described by Soyka (1956), including: a scape longer than the pedicel; the third flagellomere equal in length to the pedicel; gaster ~2× as long as wide; the longest marginal setae nearly equal to the wing width; and clava ~2× as long as F6. However, it differs in two critical aspects: the new species exhibits a forewing length-to-width ratio of 5:1 (vs ~4.6:1 in Soyka’s type specimen) and possesses a shorter mesosoma with a mesosoma-to-gaster ratio of about 0.9 (compared to ~ 0.7 in the latter).

### ﻿ Polynema (Polynema) bakkendorfi

Taxon classificationAnimaliaHymenopteraMymaridae

Hincks, 1950

E7105F20-4FC1-517B-B492-FE9713A728A8

Fig. 6

Polynema bakkendorfi Hincks, 1950: 193.

#### Material examined.

China, Xinjiang: • 1 ♀, Gongliu, 43°13'33"N, 82°43'15"E, 10.VII.2021, Qin Li et al., sweeping; • 1 ♀, Keping, 40°29'47"N, 79°08'46"E, 28.VII.2022, Qin Li et al., sweeping; • 1 ♀, Qinhe, 46°40'39"N, 90°19'58"E, 10.VII.2020, Qin Li et al., sweeping; • 1 ♀, Qinhe, 46°26'05"N, 90°02'45"E, 9.VII.2020, Qin Li et al., sweeping; • 1 ♀, Yining, 43°52'59"N, 81°22'05"E, 7.VII.2021, Qin Li et al., sweeping. (All in ICXU).

#### Diagnosis.

**Figure 6.**
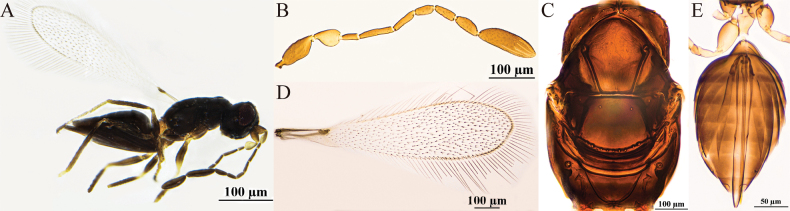
P. (Polynema) bakkendorfi ♀ (Keping, Xinjiang, China). A. Body; B. Antenna; C. Mesosoma; D. Forewing; E. Metasoma.

**Female.** Body length 1000–1100 μm (*n* = 5). Body (Fig. 6A) dark brown, scape and pedicel yellowish-brown, petiole yellowish-brown, legs dark brown. Antenna (Fig. 6B) with scape smooth; F2 the longest funicular, slightly longer than F3; F1 as long as F4 and both the shortest funiculars; clava with seven mps. Mesoscutum (Fig. 6C) with conspicuous reticulate sculpture. Forewing discal setae (Fig. 6D) originating at the lower part of the stigmal vein. Metacoxa (Fig. 6E) in lateral view almost as long as petiole.

**Male.** Unknown.

#### Hosts.

Unknown.

#### Distribution.

China (Xinjiang); United Kingdom.

#### Comments.

This species is similar to P. (Polynema) neofuscipes, both having the mesoscutum with distinctly reticulate sculpture, but in the latter, the reticulation is less pronounced. Metacoxa in lateral view of the former is shorter than the petiole, while that of the latter is as long as the petiole.

### ﻿ Polynema (Polynema) bischoffi

Taxon classificationAnimaliaHymenopteraMymaridae

(Soyka, 1950)

025C24BB-ACC3-5BE5-AB67-5386EE578607

Fig. 7

Novickyella bischoffi Soyka, 1950: 7.
Polynema bischoffi (Soyka): Trjapitzin 1978: 534.

#### Material examined.

China, Xinjiang: • 1 ♀, Fukang: 44°15'59"N, 87°32'12"E, 5.V.2017, Hongying Hu et al., sweeping; • 1 ♀, 44°13'30"N, 87°31'52"E, 23.IX.2017, Hongying Hu et al., sweeping; • 1 ♀, 44°13'20"N, 87°31'53"E, 4.VI.2017, Hongying Hu et al., sweeping. (All in ICXU).

#### Diagnosis.

**Figure 7.**
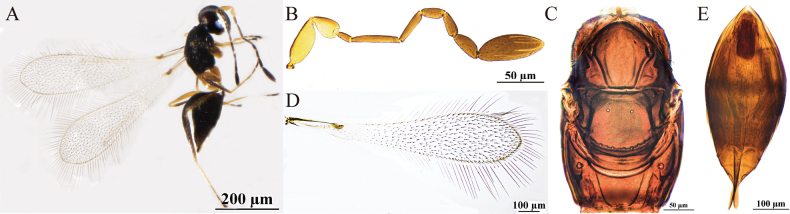
P. (Polynema) bischoffi ♀ (Fukang, Xinjiang, China). A. Body; B. Antenna; C. Mesosoma; D. Forewing; E. Metasoma.

**Female.** Body length 1090–1180 μm (*n* = 3). Body (Fig. 7A) dark brown, scape and pedicel yellowish-brown, clava dark brown, petiole yellowish-brown, legs yellowish-brown. Antenna (Fig. 7B) with scape smooth; clava shorter than combined length of 3 preceding flagellomeres, with eight mps. Mesoscutum (Fig. 7C) smooth. Propodeum (Fig. 7C) without a median carina. Forewing (Fig. 7D) longest marginal seta shorter than the greatest width of the wing. Metacoxa (Fig. 7E) and petiole in lateral view subequal in length. Ovipositor (Fig. 7E) exserted from the apex of gaster by ~0.1× its own total length.

**Male.** Unknown.

#### Hosts.

Unknown.

#### Distribution.

China (Xinjiang); Austria.

### ﻿ Polynema (Polynema) capillatum

Taxon classificationAnimaliaHymenopteraMymaridae

Soyka, 1956

3D857716-1E77-5949-B37C-7E0A1743CCEB

Fig. 8

Polynema capillata Soyka, 1956: 45.
Polynema capillatum Soyka: Trjapitzin 1978: 534.

#### Material examined.

China, Xinjiang: • 2 ♀♀, Manas, 44°13'09"N, 86°22'18"E, 22.V.202, Qin Li et al., sweeping; • ♀, Yining, 43°48'58"N, 81°28'20"E, 8.VII.2021, Qin Li et al., sweeping. (All in ICXU).

#### Diagnosis.

**Figure 8.**
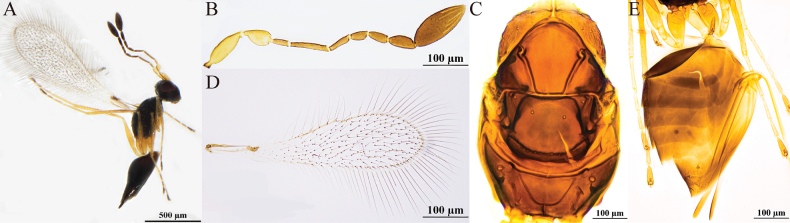
P. (Polynema) capillatum ♀ (Manas, Xinjiang, China). A. Body; B. Antenna; C. Mesosoma; D. Forewing; E. Metasoma.

**Female.** Body length 1000–1200 μm (*n* = 3). Body (Fig. 8A) dark brown, scape and pedicel yellowish-brown, clava brown; petiole and legs yellowish-brown. Antenna (Fig. 8B) with scape smooth, F4 and F5 subequal in length; clava with six mps. Pronotum (Fig. 8C) with four setae on each side along the anterior margin. Mesoscutum (Fig. 8C) smooth; propodeum (Fig. 8C) with a short median carina. Forewing (Fig. 8D) with discal cilia very long and sparse; longest marginal seta approximately as long as width of wing. Metacoxa (Fig. 8E) and petiole in lateral view subequal in length.

**Male.** Unknown.

#### Hosts.

Unknown.

#### Distribution.

China (Xinjiang); Germany, Poland.

#### Comments.

This species is similar to P. (Polynema) gracile; both have scape smooth, clava with six mps, and mesoscutum smooth. However, this species is distinguished from *P. gracile* by the pronotum with four setae on each side along the anterior margin; forewing with discal cilia being relatively sparser and with the longest marginal seta as long as width of wing. In *P. gracile*, the pronotum has seven setae on each side along the anterior margin; the forewing has very long and irregularly distributed discal setae, and the longest marginal setae are shorter than the maximum width of the wing.

### ﻿ Polynema (Polynema) ?elegantissimum

Taxon classificationAnimaliaHymenopteraMymaridae

Soyka, 1956

49192AE5-F013-55F4-8EA3-42F0A542DE80

Fig. 9

Polynema elegantissima Soyka, 1956: 48–49.
Polynema elegantissimum Soyka: Trjapitzin 1978: 536.

#### Material examined.

China, Xinjiang: • 1 ♀, Hetian, 36°54'36"N, 81°24'35"E, 3.VIII.2021, Zhulidezi Aishan et al., sweeping; • 1 ♀, Ruoqiang, 38°40'05"N, 87°18'16"E, 29.VII.2021, Zhulidezi Aishan et al., sweeping. (All in ICXU).

#### Diagnosis.

**Figure 9.**
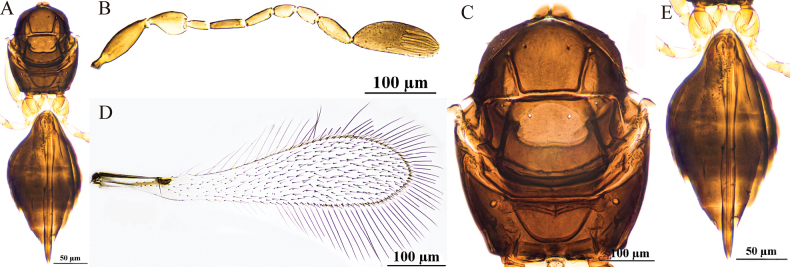
P. (Polynema) ? elegantissimum ♀ (Ruoqiang, Xinjiang, China). A. Habitus in dorsolateral view; B. Antenna; C. Mesosoma; D. Forewing; E. Metasoma.

**Female.** Body length 700–800 μm (*n* = 2). Body (Fig. 9A) dark brown, scape and pedicel yellow, clava dark brown, petiole yellowish-brown, legs pale brown. Antenna (Fig. 9B) with scape smooth, clava longer than combined length of three preceding flagellomeres, with eight mps. Mesoscutum (Fig. 9C) with indistinct reticulate sculpture. Propodeum (Fig. 9C) without a median carina. Forewing (Fig. 9D) with longest marginal seta 0.9–1.3× as long as the greatest width of the wing; forewing with hypochaeta extending to posterior margin of forewing. Metacoxa (Fig. 9E) and petiole in lateral view subequal in length. Ovipositor (Fig. 9E) exserted from gastral gaster by 0.1× its own total length.

**Male.** Unknown.

#### Hosts.

Unknown.

#### Distribution.

China (Xinjiang); Greece, Netherlands.

#### Comments.

The features of these tentatively identified specimens from Xinjiang are similar to those described by Soyka (1956); however, there are differences between them, as follows: the body color in the specimens from Xinjiang is dark brown whereas Soyka described his specimens as pale yellow-brown (however, that could be due to slide-mounting); the clava length is longer than the combined length of F1–F3 and also longer than the combined length of F4–F6 in our specimens, whereas in Soyka’s description, the length of the clava is shorter than the combined length of F1–F3 and slightly shorter than the combined length of F4–F6.

### ﻿ Polynema (Polynema) elongatum

Taxon classificationAnimaliaHymenopteraMymaridae

Soyka, 1956

5F6F9EE3-962C-5BF9-AEB6-D10509032B13

Fig. 10

Polynema elongata Soyka, 1956: 48–49.
Polynema elongatum Soyka: Trjapitzin 1978: 536.

#### Material examined.

China, Xinjiang: • 2 ♀♀, Hetian: 36°21'3"N, 81°27'03"E, 12.VII.2021, Zhulidezi Aishan et al., sweeping; • 1 ♀, 36°54'36"N, 81°24'35"E, 3.VIII.2021, Zhulidezi Aishan et al., sweeping; • 1 ♀, Kashi, 39°24'35"N, 76°12'36"E, 8.VIII.2021, Zhulidezi Aishan et al., sweeping. (All in ICXU).

#### Diagnosis.

**Figure 10.**
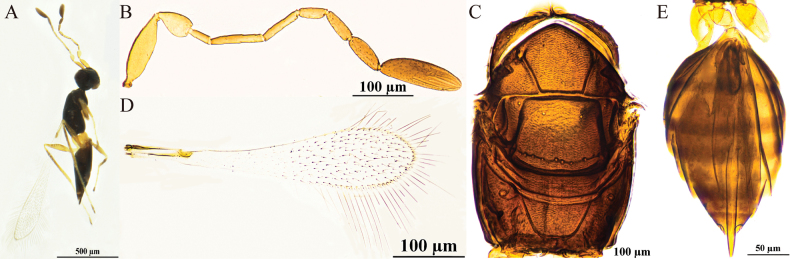
P. (Polynema) elongatum ♀ (Hetian, Xinjiang, China). A. Body; B. Antenna; C. Mesosoma; D. Forewing; E. Metasoma.

**Female.** Body length 860–910 μm (*n* = 4). Body (Fig. 10A) dark brown, scape and pedicel yellow, clava pale brown, petiole yellow, legs yellowish-brown. Antenna (Fig. 10B) with scape smooth, longer than pedicel, 1.9–2.3× as long as pedicel; clava with seven mps; pronotum with eight setae on each side along the anterior margin (Fig. 10C); propodeum (Fig. 10C) with a short median carina. Forewing (Fig. 10D) 5.0× as long as wide, the longest marginal seta as long as the greatest width of wing. Ovipositor (Fig. 10E) exserted beyond apex of gaster by ~0.1× its own total length; ovipositor 1.4–1.5× length of metatibia.

**Male.** Unknown.

#### Hosts.

Unknown.

#### Distribution.

China (Xinjiang); Netherlands.

#### Comments.

The specimens from Xinjiang have eight setae on each side along the anterior margin of the pronotum. Other, more or less similar, species typically have 4–6 such setae.

### ﻿ Polynema (Polynema) fumipenne

Taxon classificationAnimaliaHymenopteraMymaridae

Walker, 1846

EFC31516-0674-504F-9AEB-50875D344650

Fig. 11

Polynema fumipennis Walker, 1846: 52.
Polynema fumipenne Walker: Boţoc 1963: 104.

#### Material examined.

China, Xinjiang: • 1 ♀, Qinhe, 46°40'39"N, 90°19'58"E, 10.VII.2020, Qin Li et al., sweeping; • 1 ♀, Xinyuan, 43°13'24"N, 83°21'17"E, 8.VIII.2017, Hongying Hu et al., sweeping. (All in ICXU).

#### Diagnosis.

**Figure 11.**
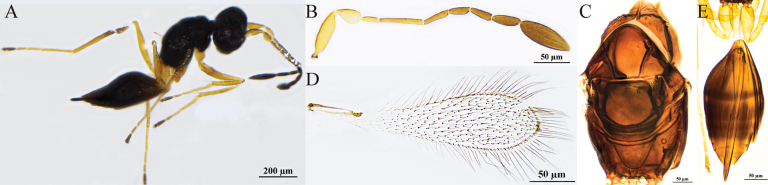
P. (Polynema) fumipenne ♀ (Qinhe, Xinjiang, China). A. Body; B. Antenna; C. Mesosoma; D. Forewing; E. Metasoma.

**Female.** Body length 1130–1165 μm (*n* = 2). Body (Fig. 11A) dark brown, scape and pedicel yellow, clava pale brown, petiole and legs yellow. Antenna (Fig. 11B) with scape smooth; F1 as long as F4 and both the longest funiculars; clava with six mps. Mesoscutum (Fig. 11C) smooth; pronotum with four setae on each side along the anterior margin (Fig. 11C); propodeum (Fig. 11C) without a median carina. Forewing (Fig. 11D) with discal cilia extending to approximately mid-length of submarginal vein. Metacoxa (Fig. 11E) in lateral view 0.7× as long as petiole. Ovipositor (Fig. 11E) slightly exserted from the apex of gaster.

**Male.** Unknown.

#### Hosts.

Unknown.

#### Distribution.

China (Xinjiang); Europe.

#### Comments.

These specimens from Xinjiang show are very similar to the European *P. fumipenne* in most morphological features. However, in the former the metacoxa is 0.7× as long as petiole, whereas in the European specimens it is slightly longer than the petiole.

### ﻿ Polynema (Polynema) ?gracile

Taxon classificationAnimaliaHymenopteraMymaridae

(Nees, 1834)

FDDA93C6-FBE0-5B42-8149-F18CF36A6D80

Fig. 12

Eutriche gracilis Nees, 1834: 197. Walker 1846: 52; Foerster 1847: 217–218; Hincks 1950: 181.
= Polynema ovulorum L. (synonymy Graham 1973).
Polynema britteni Hincks, 1950: 185. Synonymy by Graham 1973: 363.
Polynema gracile (Nees): Graham 1973: 363.
Polynema (Polynema) gracile (Nees): Triapitsyn and Fidalgo 2006: 60–61; Schuppenhauer and Triapitsyn 2018: 162, 166–167.

#### Material examined.

• 1 ♀ (ICXU), China, Xinjiang, Xinyuan, 43°13'24"N, 83°21'17"E, 8.VIII.2017, Hongying Hu et al., sweeping.

#### Diagnosis.

**Figure 12.**
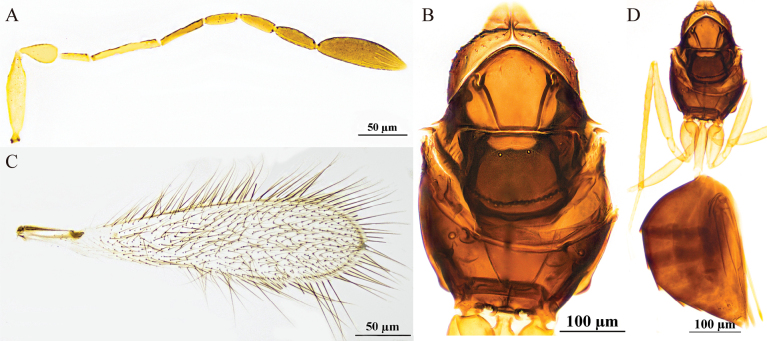
P. (Polynema) ? gracile ♀ (Xinyuan, Xinjiang, China). A. Antenna; B. Mesosoma; C. Forewing; D. Habitus in dorsolateral view (metasoma).

**Female.** Body length 1200μm (*n* = 1). Antenna (Fig. 12A) with scape smooth, 2.5× as long as pedicel; clava with six mps. Mesoscutum (Fig. 12B) smooth. Forewing with discal microtrichia (Fig. 12C) very long, irregularly arranged and extending proximally to approximately mid-length of submarginal vein. Metacoxa (Fig. 12D) in lateral view shorter than petiole; ovipositor (Fig. 12D) barely protruding beyond apex of gaster.

**Male.** Unknown.

#### Hosts.

Unknown.

#### Distribution.

China (Xinjiang); Europe.

#### Comments.

The morphological characteristics of this specimen align closely with Schuppenhauer and Triapitsyn’s (2018) redescription of *P. gracile*, sharing key features including consistent body coloration, similar body length (1200–1400 μm vs 1200–1390 μm), a smooth antennal scape, F2 being the longest funicular segment, ventral sickle-shaped sensilla on F5 and F6, a short propodeal median carina, and a slightly exposed ovipositor. Minor variations include the mesoscutum being smooth (lacking the faint reticulation noted by Soyka), the clavomeres bearing six mps instead of seven (likely due to observational variation), and a slightly higher ratio of longest marginal fringe seta to maximum wing width (0.8 vs 0.63–0.71 in the European specimens). Given the fundamental consistency in the core traits – coloration, antennal segmentation pattern, propodeal structure, forewing proportions, etc. –and the presence of some minor differences, this specimen is tentatively identified as *P. gracile*.

### ﻿ Polynema (Polynema) ?neofuscipes

Taxon classificationAnimaliaHymenopteraMymaridae

(Soyka, 1950)

5FD4DA9F-D8E3-5703-A920-318BFEE44216

Fig. 13

Maidliella neofuscipes Soyka, 1946b: 178.
Polynema neofuscipes (Soyka): Trjapitzin 1978: 533.
Polynema (Polynema) neofuscipes (Soyka): Triapitsyn and Fidalgo 2006: 61.

#### Material examined.

China, Xinjiang: • 1 ♀, Huocheng, 43°58'00"N, 80°52'55"E, 5.VII.2021, Qin Li et al., sweeping; • 1 ♀, Shaya, 41°44'24"N, 82°45'03"E, 15.VIII.2021, Qin Li et al., sweeping; •1 ♀, Yining, 43°52'59"N, 81°22'05"E, 7.VII.2021, Qin Li et al., sweeping. (All in ICXU).

#### Diagnosis.

**Figure 13.**
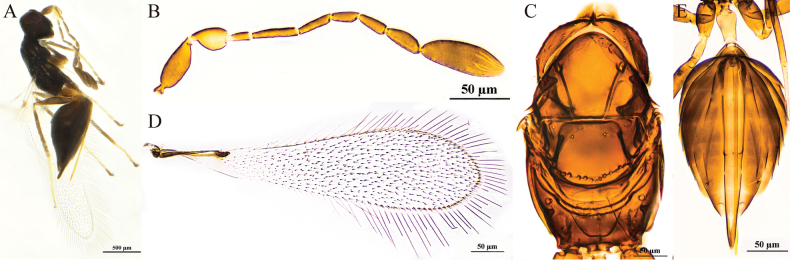
P. (Polynema) ? neofuscipes ♀ (Yining, Xinjiang, China). A. Body; B. Antenna; C. Mesosoma; D. Forewing; E. Metasoma.

**Female.** Body length 870–1060 μm (*n* = 3). Body (Fig. 13A) dark brown, scape and clava pale brown, pedicel yellowish-brown; petiole and legs pale brown. Antenna (Fig. 13B) with scape with indistinct cross-ridges, clava with seven mps. Propodeum (Fig. 13C) with a short median carina. Forewing (Fig. 13D) 3.9–4.0× as long as wide; longest marginal seta 0.6–0.8× as long as the greatest width of wing, with discal microtrichia short, uniformly arranged; dark microtrichia on disc beginning behind stigmal vein. Metacoxa (Fig. 13E) in lateral view shorter than petiole.

**Male.** Unknown.

#### Hosts.

Unknown.

#### Distribution.

China (Xinjiang); Netherlands.

#### Comments.

The morphological characteristics of this specimen show a high degree of consistency with Soyka’s (1946) original description of *P. neofuscipes*. Key shared diagnostic features include: identical body coloration; scape with transverse striations; F2 being the longest funicular segment while F1 is the shortest; mesoscutum with reticulate sculpture; propodeum bearing a relatively short median carina; and conspicuously protruding ovipositor. Minor variations observed include: forewing length-to-width ratio of 3.7–4.1 in this specimen vs 4.5 in the original description; clavomeres bearing seven mps compared to eight in the type specimen (likely due to observational differences); and the longest marginal fringe seta to maximum wing width ratio being 0.7–0.9× vs 0.64× in the original description. Considering these relatively minor discrepancies in conjunction with the fundamental consistency in all critical diagnostic characteristics, this specimen is provisionally identified as *P. neofuscipes*.

### ﻿ Polynema (Polynema) pusillum

Taxon classificationAnimaliaHymenopteraMymaridae

Haliday, 1833

EE2B7CBB-4AA7-5EA9-882D-C9658D4B3DA4

Fig. 14

Polynema pusillus Haliday, 1833b: 349.
Cosmocoma pusilla (Haliday): Marshall 1873: 24.
Polynema (Polynema) pusillum Haliday: Triapitsyn and Fidalgo 2006: 61; Schuppenhauer and Triapitsyn 2018: 168, 170–171.

#### Material examined.

China, Xinjiang: • 1 ♀, Huocheng, 43°56'41"N, 80°52'13"E, 5.VII.2021, Qin Li et al., sweeping; • 1 ♀, Qinhe, 46°26'05"N, 90°02'45"E, 9.VII.2020, Qin Li et al., sweeping; • 1 ♀, Tacheng, 46°36'34"N, 82°57'59"E, 29.VI.2021, Qin Li et al., sweeping. (All in ICXU).

#### Diagnosis.

**Figure 14.**
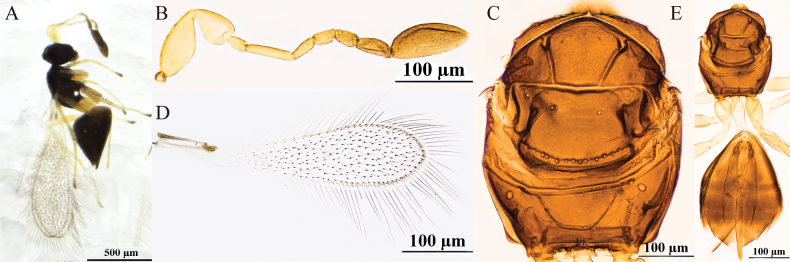
P. (Polynema) pusillum ♀ (Qinhe, Xinjiang, China). A. Body; B. Antenna; C. Mesosoma; D. Forewing; E. Habitus in dorsolateral view (metasoma).

**Female.** Body length 710–800 μm (*n* = 3). Body (Fig. 14A) dark brown, scape and pedicel yellow, F1–F5 pale yellow, F6 and clava brown; petiole and legs yellow. Antenna (Fig. 14B) with scape smooth, 2.5–2.7× as long as wide (including short radicle); F4 and F5 subequal in length; clava as long as combined length of three preceding flagellomeres, with seven mps. Mesoscutum (Fig. 14C) with indistinct reticulate sculpture. Metacoxa (Fig. 14E) in lateral view 1.1–1.2× as long as petiole.

**Male.** Unknown.

#### Hosts.

Unknown.

#### Distribution.

China (Xinjiang); Europe and Turkey.

#### Comments.

The specimens from Xinjiang share similarities with the European specimens of *P. pusillum*, as redescribed by Schuppenhauer and Triapitsyn (2018), in having a short median carina on the propodeum and the longest marginal setae on the forewing are ~0.7× the wing width. However, they differ in that the scape of the Xinjiang specimens is smooth, whereas the scape of the European *P. pusillum* has some faint longitudinal striations.

## ﻿Discussion

*Polynema* is one of several speciose cosmopolitan genera of Mymaridae and is common in the Palearctic region (Triapitsyn 2017; UCD 2025). This study reports 11 newly recorded species in China, some of doubtful identity. Our findings indicate that several *Polynema* species have much wider distributions in the Palearctic region than previously known. This is not surprising as Xinjiang, a Central Asian area, has a temperate to fairly cold climate, similar to that of Europe, and also a wide range of altitudes. The species recorded here were collected from 376–1700 m. With further collecting in the intervening regions, it is quite likely that more species of Mymaridae will be found to occur not only in Xinjiang but across the entire Palearctic region. An example is the discovery of P. (Polynema) fumipenne in Xinjiang which aligns with its widespread distribution across Europe, from the United Kingdom to western Russia. The species composition in Xinjiang has not been compared with that of the neighboring Central Asian countries (e.g., Kazakhstan), from which almost no Mymaridae have been known. Future collecting across central Asia will undoubtedly help clarify the distribution patterns of these species.

No hosts have been recorded for the *Polynema* species in China. Literature records indicate that species of P. (Polynema) are egg parasitoids of Cicadellidae and Miridae (Hemiptera) (Huber et al. 2020). The collection site of *P. haloxyloniae* (desert low-altitude area) overlaps with the distribution of its host plants *Haloxylon* spp., so it could be a specialist parasitoid of an unknown host on this plant genus with often specialized insect fauna. The *Polynema* species collected in this study from Xinjiang were distributed across a variety of habitats including desert shrublands (e.g., *Haloxylon* forests), montane grasslands, and river valley oases. This altitudinal range corresponds closely with Xinjiang’s distinctive topographic gradient, which includes the desert–oasis ecotone of the Tarim Basin (elevation <1000 m), temperate grasslands on the northern slopes of the Tianshan Mountains (1000–2000 m), and the humid forest margins of the Ili River Valley (500–1500 m). Notably, *P. atrosimile* and *P. bakkendorfi* were collected across a broader elevational range of 515–1136 m, indicating a wider ecological tolerance for mid-elevation habitats.

## Supplementary Material

XML Treatment for Polynema (Polynema)

XML Treatment for Polynema (Polynema) breviscapus

XML Treatment for Polynema (Polynema) gongliuense

XML Treatment for Polynema (Polynema) haloxyloniae

XML Treatment for Polynema (Polynema) antoniae

XML Treatment for Polynema (Polynema) atrosimile

XML Treatment for Polynema (Polynema) bakkendorfi

XML Treatment for Polynema (Polynema) bischoffi

XML Treatment for Polynema (Polynema) capillatum

XML Treatment for Polynema (Polynema) ?elegantissimum

XML Treatment for Polynema (Polynema) elongatum

XML Treatment for Polynema (Polynema) fumipenne

XML Treatment for Polynema (Polynema) ?gracile

XML Treatment for Polynema (Polynema) ?neofuscipes

XML Treatment for Polynema (Polynema) pusillum

## ﻿Additional information

### Use of AI

No use of AI was reported.

### Author ORCIDs

Zhulidezi Aishan ￼ https://orcid.org/0000-0003-2712-4049

Serguei V. Triapitsyn ￼ https://orcid.org/0000-0002-5086-7847

### Data availability

All of the data that support the findings of this study are available in the main text.
